# Basic psychological needs and psychache: associations with cognitive reappraisal and stress mindset in a cross-sectional sample

**DOI:** 10.3389/fpsyg.2026.1783720

**Published:** 2026-04-10

**Authors:** Yangkun Hou, Honggang Li, Weiwei Ma, Jinxia Wu, Yufu Wang, Jianlan Liu

**Affiliations:** 1 School of Clinical Medicine, Shandong Second Medical University, Weifang, Shandong, China; 2 Student Affairs Office of Shandong Second Medical University, Weifang, Shandong, China; 3 School of Health Management, Qingdao Hengxing University of Science and Technology, Qingdao, Shandong, China

**Keywords:** basic psychological needs, cognitive reappraisal, medical students, psychache, stress mindset

## Abstract

**Introduction:**

This study examined the associations between basic psychological needs (BPN) and psychache among medical students and tested whether cognitive reappraisal statistically accounted for part of this association and whether these associations varied across levels of stress mindset.

**Methods:**

A cross-sectional survey was conducted among medical students from five medical schools in Shandong Province, China. A total of 2,157 valid questionnaires were included in the analyses. Structural equation modeling was used to test the hypothesized associations.

**Results:**

Higher BPN was associated with lower psychache. Higher BPN was also associated with greater cognitive reappraisal, which in turn was associated with lower psychache. The indirect association via cognitive reappraisal was significant at low and average levels of stress mindset, but not at high levels. In addition, the negative associations of both BPN and cognitive reappraisal with psychache were weaker at higher levels of stress-is-enhancing mindset.

**Discussion:**

These findings suggest that motivational resources, emotion regulation, and stress-related beliefs should be considered jointly when examining psychache in medical students. Because all variables were assessed at one time point in a geographically restricted and educationally homogeneous sample, the findings should be interpreted as context-specific cross-sectional associations rather than evidence of temporal or causal processes.

## 1 Introduction

### 1.1 Basic psychological needs and psychache

Psychache is a clinically important form of psychological distress that reflects intense emotional pain and has been linked to suicidal risk, impaired functioning, and poor mental health outcomes (Shneidman, 1998; Baryshnikov and Isometsä, 2022). Medical students may be especially vulnerable because they face heavy academic workloads, repeated evaluation, and demanding clinical preparation, all of which can heighten psychological strain (Mao et al., 2019; Hawsawi et al., 2025). Given the implications of psychache for both student well-being and professional functioning, identifying correlates and potential protective factors is an important goal in medical education research.

Self-Determination Theory (SDT) provides one relevant framework for understanding why psychache may vary across students. SDT proposes that well-being depends in part on satisfaction of three basic psychological needs: autonomy, competence, and relatedness (Deci and Ryan, 2004). When these needs are met, individuals typically report better adjustment and psychological functioning; when they are frustrated, distress is more likely. Prior work has linked lower need satisfaction to anxiety, depression, loneliness, and other maladaptive outcomes (Neufeld and Malin, 2019; Bai et al., 2021; Jovanović et al., 2024). On this basis, we expected BPN fulfillment to show a negative association with psychache among medical students.

### 1.2 Cognitive reappraisal in the association between BPN and psychache

BPN may also be relevant to psychache through emotion regulation. According to Gross’s process model, cognitive reappraisal is an antecedent-focused strategy that changes how a situation is interpreted before emotional responses fully unfold (Gross, 2015). Compared with less adaptive strategies such as suppression, reappraisal is generally associated with lower negative affect, better interpersonal functioning, and fewer psychological symptoms (Goldin et al., 2008; Wu et al., 2019; Chen et al., 2023).

SDT suggests that students whose needs for autonomy, competence, and relatedness are better met should have greater psychological resources to engage adaptive self-regulation. Consistent with this idea, need-supportive contexts and greater need fulfillment have been linked to more adaptive coping and more positive emotional functioning, including greater use of reappraisal (Robazza et al., 2023). In turn, more frequent use of reappraisal may be associated with lower psychache because stressful experiences are interpreted in less threatening ways. Accordingly, we examined whether cognitive reappraisal statistically accounted for part of the association between BPN and psychache in the present cross-sectional data.

### 1.3 Stress mindset as a boundary condition of associations

Stress mindset may also be associated with variation in the strength of these associations. Stress mindset refers to beliefs about whether stress is primarily enhancing or debilitating for performance, learning, and health (Crum et al., 2013). Individuals with a stronger stress-is-enhancing mindset tend to interpret stress more constructively and often show more adaptive cognitive, emotional, and behavioral responses under challenge (Crum et al., 2017; Zeng et al., 2024).

This construct is especially relevant here because it may be associated with how strongly BPN and reappraisal relate to psychache. A more stress-is-enhancing mindset may be associated with lower psychache overall and with weaker negative associations of BPN and reappraisal with psychache. We therefore conceptualized stress mindset as a boundary condition of the BPN–psychache and reappraisal–psychache associations rather than as evidence of a buffering mechanism.

Few studies have examined BPN, cognitive reappraisal, stress mindset, and psychache within a single model. The present study addresses this gap by integrating Self-Determination Theory with the process model of emotion regulation in a cross-sectional sample of medical students. In this framework, BPN represents a motivational context, cognitive reappraisal represents an adaptive regulatory correlate, and stress mindset represents a belief-based condition that may be associated with variation in the strength of observed associations with psychache.

Specifically, we examined whether higher BPN was associated with lower psychache, whether cognitive reappraisal statistically accounted for part of this association, and whether the associations of BPN and reappraisal with psychache varied across levels of stress mindset. Because the study was cross-sectional, any indirect effect was interpreted as statistical mediation only, not as evidence of temporal sequencing or causal mechanism. The goal was therefore theory-informed, hypothesis-generating clarification rather than causal explanation.

The study focused on medical students from five medical schools in Shandong Province, China. Given the regional and educational boundaries of the sample, the findings are best interpreted as context-specific and should not be generalized broadly beyond similar settings.

**Figure 1:**
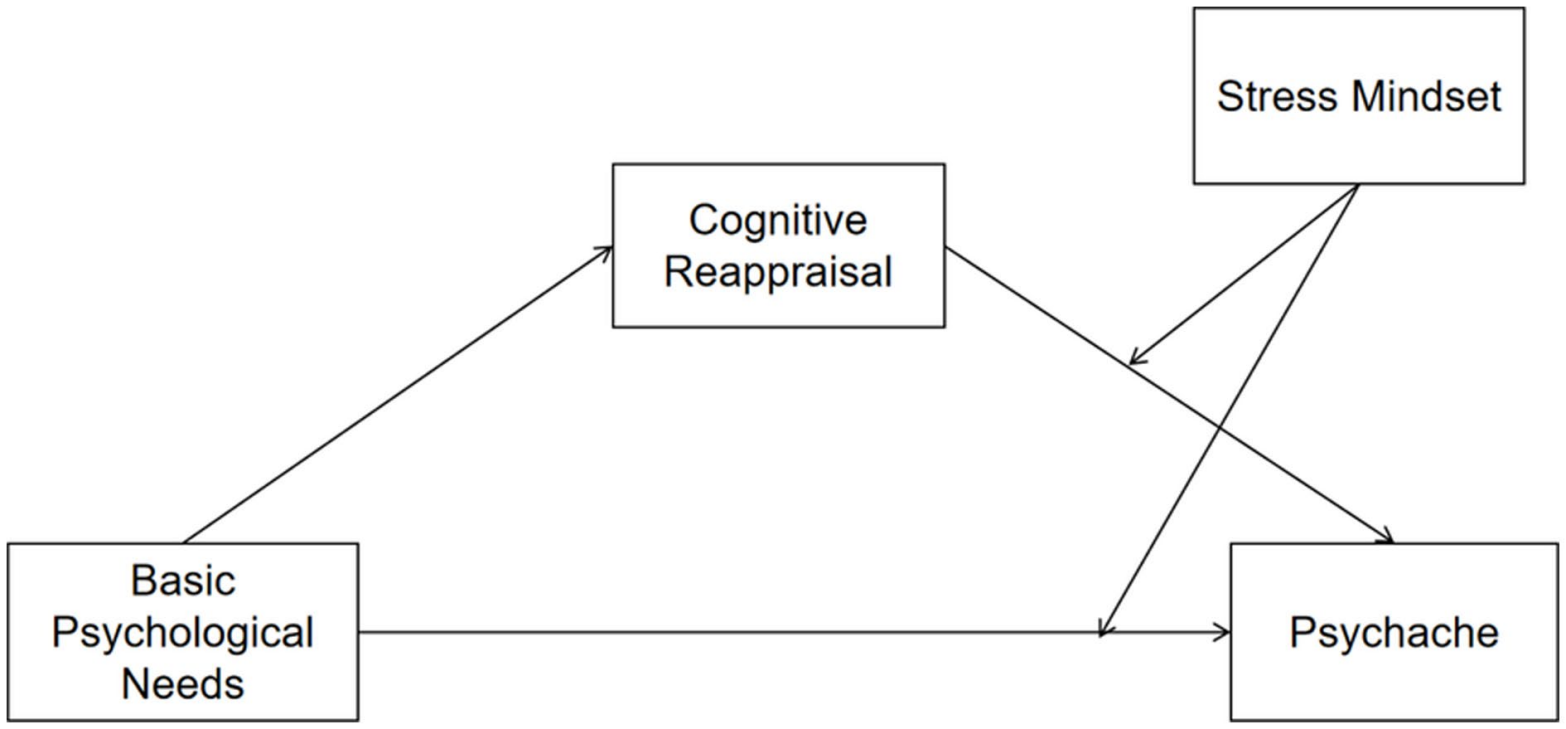
Hypothesized associations among BPN, cognitive reappraisal, stress mindset, and psychache.

Based on these considerations, we propose the following hypotheses (Figure 1):

*Hypothesis 1*: Medical students’ BPN is negatively associated with their psychache.

*Hypothesis 2*: Cognitive reappraisal statistically accounts for the association between BPN and psychache.

*Hypothesis 3*: The association between BPN and psychache varies across levels of stress mindset.

*Hypothesis 4*: The association between cognitive reappraisal and psychache varies across levels of stress mindset.

## 2 Methodology

### 2.1 Research design

This study used a cross-sectional questionnaire design. Data were collected anonymously in classroom settings between April and May 2024 to examine associations among psychache, basic psychological needs, cognitive reappraisal, and stress mindset in medical students.

### 2.2 Participants and sampling

We used a convenience-within-cluster sampling strategy. Five medical schools in Shandong Province were selected based on feasibility and to improve coverage across the province, but the sample should not be considered geographically representative. Within each selected school, classes were sampled by cluster after class rosters for different grades had been obtained, and all students in the selected classes were invited to participate.

A total of 2,633 questionnaires were collected. After excluding incomplete responses, patterned answers, or invalid responses failing attention-check items (e.g., “Please select ‘Strongly Agree’”), 2,157 valid samples were obtained, yielding an effective response rate of 81.9%. (Table 1).

**Table 1:** Participants’ demographic characteristics (*N* = 2,157).

Variables	Category	Frequency	Percentage (%)
Gender	Male	767	35.56
	Female	1,390	64.44
Only child	Yes	690	31.99
	No	1,467	68.01
Source of students	City	965	44.74
	Village	1,192	55.26
Educational system	Four-year program	479	22.21
	Five-year program	1,678	77.79
Grade	Freshman	1,108	51.36
	Sophomore	310	14.37
	Junior	425	19.70
	Senior	126	5.84
	Fifth-year	131	6.07
	Postgraduate	57	2.64
Parents are in the medical profession	Neither	1870	86.69
	One	209	9.69
	Both	78	3.62

### 2.3 Measures and procedures

Data were collected using an anonymous questionnaire that included standardized measures of psychache, basic psychological needs, stress mindset, and cognitive reappraisal. The protocol was approved by the Ethics Committee of Shandong Second Medical University. Trained research assistants administered the survey in classrooms using a standardized script that emphasized voluntariness, anonymity, confidentiality, and the right to withdraw at any time. Completion took approximately 30 min.

#### 2.3.1 Procedural remedies for common method bias

To reduce the likelihood of common method bias, several procedural remedies recommended by Podsakoff et al. (2003) were implemented during questionnaire design and administration. Specifically, different response formats and scale anchors were used across measures, participants were assured of anonymity and confidentiality, and validated instruments with clear wording were adopted. In addition, the order of the scales was randomized to minimize systematic order effects and method-driven responding.

### 2.4 Research instruments

Psychache was measured using the Psychache Scale (PAS), which was developed by Holden et al. (2001). Qin (2008) localized the scale in 2008. The scale consists of 13 items, scored on a 5-point Likert scale. The total score can range from 13 to 65. The higher the score, the more intense the psychache experienced. The Cronbach’s alpha for this study was 0.97.

The Basic Psychological Needs Scales (BPNS; Gagné, 2003) developed by Gagne et al. in 2003 was used to measure the BPNS. Different countries have used this questionnaire with satisfactory reliability, finding it applicable to people of different ages, groups, and cultural backgrounds. Liu Junsheng et al. translated and localized the Chinese version, showing excellent reliability and validity with an internal consistency coefficient of 0.88 (Liu et al., 2013). The questionnaire consisted of 21 items scored on a 7-point Likert scale. Nine of these items underwent reverse scoring. Higher scores indicate a higher degree of realization of an individual’s psychological needs. The Cronbach’s *α* for this study was 0.92.

The Stress Mindset Measure (SMM) developed by Crum et al. (2013) was used to measure stress mindset. Our scholar He et al. (2022) localized this scale. Subjects rated each of the eight items on a five-point scale (0 = strongly disagree, 4 = strongly agree). The items in the stress negative affect dimension are reverse-scored items with a score range of 0–32. The higher the score, the more the individual tends to perceive stressful influences as positive; the lower the score, the more the individual tends to perceive stressful influences as negative. The Cronbach’s *α* of the questionnaire was tested to be 0.79.

The Emotion Regulation Questionnaire developed by Gross and John (2003) was used, which has excellent reliability and validity and includes two dimensions, expression inhibition and cognitive reappraisal, with 10 items. The cognitive reappraisal dimension is associated with six of these items. This study utilized the questionnaire’s cognitive reappraisal scale. The scale is scored on a 7-point Likert scale and includes questions such as “I often control my emotions by changing the way I analyze and perceive my environment” and other questions. The scale reflects how often individuals use cognitive reappraisal in their daily lives. The higher the score, the more individuals tend to use cognitive reappraisal strategies. The internal consistency coefficient for this study was 0.85.

### 2.5 Data processing and analysis

Data screening and descriptive analyses were conducted in SPSS 27.0. We examined means, standard deviations, and bivariate correlations, and invalid questionnaires were removed during screening. Because questionnaires with substantial missing or invalid responses were excluded before analysis, the SEM models were estimated on complete cases. To facilitate interpretation of interaction terms and conditional effects, composite scores for BPN, psychache, cognitive reappraisal, and stress mindset were z-standardized prior to model estimation. Before fitting the structural models, we also inspected the distributions of the composite variables and correlations among predictors to check for major anomalies that would complicate estimation or suggest problematic multicollinearity. SEM analyses were conducted in Mplus 8.3 with gender and only-child status included as controls. Indirect associations were evaluated using 10,000 bootstrap resamples and bias-corrected 95% confidence intervals.

#### 2.5.1 Statistical assessment of common method bias

In addition to the procedural remedies, we conducted statistical diagnostics to evaluate the potential influence of common method bias. First, Harman’s single-factor test showed that the first unrotated factor accounted for 28.7% of the total variance, which was below the conventional 50% threshold, suggesting that no single factor dominated the covariance among the study variables. Second, an unmeasured latent method construct (ULMC) approach was applied by adding a latent common method factor to the measurement model. The improvement in model fit was small (ΔCFI = 0.012, ΔTLI = 0.011), and the substantive factor loadings remained significant with only minor changes. Taken together, these procedural and statistical checks provide some reassurance, but they do not rule out common method bias. Given the single-source, single-time-point self-report design, shared method variance remains a plausible alternative explanation and should be considered when interpreting the findings.

## 3 Results

### 3.1 Descriptive statistics and correlation analysis

Descriptive statistics and Pearson correlations are presented in Table 2. The correlational pattern was consistent with the hypothesized model. BPN showed a moderate negative association with psychache (*r* = −0.513, *p* < 0.001). Cognitive reappraisal was positively associated with BPN (*r* = 0.396, *p* < 0.001) and negatively associated with psychache (*r* = −0.279, *p* < 0.001). Stress mindset was positively associated with BPN (*r* = 0.405, *p* < 0.001) and cognitive reappraisal (*r* = 0.218, *p* < 0.001), and negatively associated with psychache (*r* = −0.296, *p* < 0.001). These correlations indicate meaningful but not redundant overlap among the constructs and provide initial support for the subsequent SEM analyses.

**Table 2:** Degree of correlation between variables.

Variables	M	SD	Psychache	BPN	Stress mindset	Cognitive reappraisal
Psychache	20.16	11.938	1			
BPN	107.44	20.074	−0.513^***^	1		
Stress mindset	17.50	4.189	−0.296^***^	0.405^***^	1	
Cognitive reappraisal	30.71	7.015	−0.279^***^	0.396^***^	0.218^***^	1

*** . At the 0.001 level (two-tailed), the correlation was significant.

### 3.2 Test of mediating effects

To test whether cognitive reappraisal statistically accounted for the association between BPN and psychache, we estimated conditional indirect effects with 10,000 bootstrap resamples while controlling for gender and only-child status. Higher BPN was associated with greater cognitive reappraisal (*b* = 0.394, *p* < 0.001), and the association between cognitive reappraisal and psychache depended on stress mindset (*b* = −0.075, *p* < 0.001; cognitive reappraisal × stress mindset: *b* = 0.054, *p* = 0.005). The indirect association (BPN → cognitive reappraisal → psychache) was significant when stress mindset was low (−1 SD; IND = −0.051, 95% CI [−0.075, −0.026]) and at the mean level (IND = −0.030, 95% CI [−0.044, −0.015]), but not at high stress mindset (+1 SD; IND = −0.008, 95% CI [−0.025, 0.008]). In substantive terms, reappraisal accounted for a modest portion of the BPN–psychache association, and this contribution diminished as stress mindset became more stress-is-enhancing.

### 3.3 Test of a moderated mediation model

The moderated mediation model showed acceptable fit, *χ*^2^(3) = 24.147, RMSEA = 0.059 (90% CI [0.038, 0.081]), CFI = 0.981, TLI = 0.918, and SRMR = 0.022. As shown in Table 3, BPN was positively associated with cognitive reappraisal (b = 0.394, *p* < 0.001). At the mean level of stress mindset, both BPN (*b* = −0.427, *p* < 0.001) and cognitive reappraisal (*b* = −0.075, *p* < 0.001) were negatively associated with psychache. The model explained 15.9% of the variance in cognitive reappraisal and 30.4% of the variance in psychache, indicating modest explanatory power.

**Table 3:** Structural equation model results (moderated mediation model).

Outcome	Predictor	*b*	SE	*z*	*p*	*β* (STDYX)
Panel A. Path coefficients (unstandardized and standardized)						
Cognitive reappraisal	BPN	0.394	0.021	18.762	<0.001	0.394
	Gender	−0.084	0.044	−1.909	0.055	−0.040
	Only-child	0.020	0.029	0.690	0.490	0.015
Psychache	Cognitive reappraisal	−0.075	0.019	−3.947	<0.001	−0.075
	Stress mindset	−0.125	0.021	−5.952	<0.001	−0.125
	Cognitive reappraisal × stress mindset (INT1)	0.054	0.019	2.842	0.005	0.058
	BPN	−0.427	0.024	−17.792	<0.001	−0.427
	BPN × stress mindset (INT2)	0.106	0.019	5.579	<0.001	0.125
	Gender	−0.095	0.041	−2.317	0.022	−0.045
	Only-child	−0.008	0.027	−0.296	0.764	−0.006

Effect	−1 SD (low)	95% CI	Mean (0)	95% CI	+1 SD (high)	95% CI
Panel B. Conditional effects and index of moderated mediation (bootstrap 95% CI)						
Conditional indirect effect (IND): BPN → cognitive reappraisal → psychache	−0.051	[−0.075, −0.026]	−0.030	[−0.044, −0.015]	−0.008	[−0.025, 0.008]
Conditional direct effect (DIR): BPN → psychache	−0.532	[−0.599, −0.469]	−0.427	[−0.475, −0.378]	−0.321	[−0.376, −0.265]
Conditional total effect (TOT)	−0.583	[−0.642, −0.527]	−0.456	[−0.501, −0.412]	−0.329	[−0.385, −0.272]

Index of moderated mediation (IMM) = 0.021, 95% CI [0.007, 0.036]. BPN, basic psychological needs; CR, cognitive reappraisal. INT1, cognitive reappraisal × stress mindset; INT2, BPN × stress mindset. Cognitive reappraisal and stress mindset were *z*-standardized prior to creating interaction terms. Estimates are unstandardized b unless otherwise noted; *β*-values are STDYX standardized estimates from Mplus. Bootstrap confidence intervals were based on 10,000 resamples.

**Figure 2:**
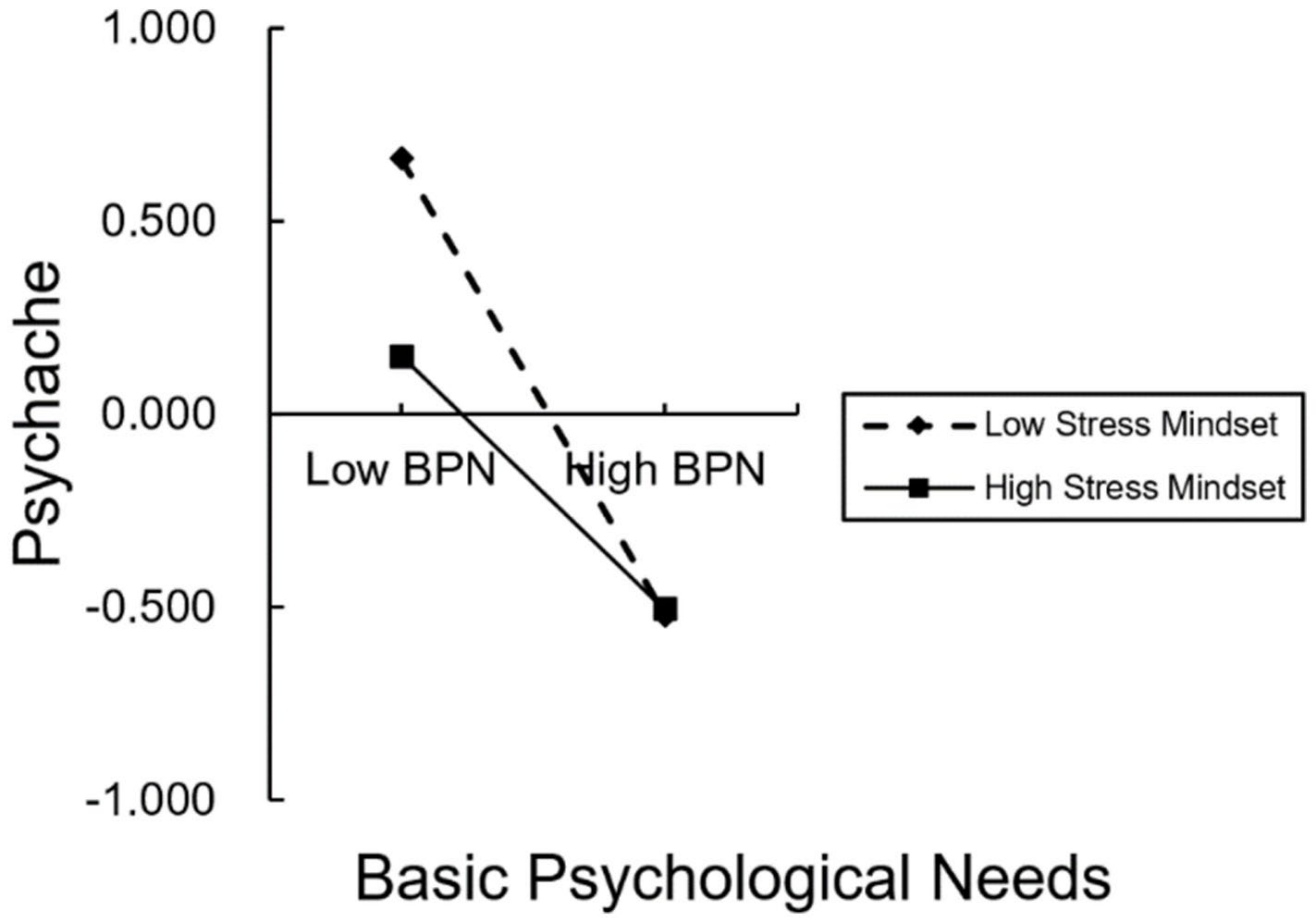
Conditional association between BPN and psychache at low (−1 SD) and high (+1 SD) levels of stress mindset.

A significant interaction between BPN and stress mindset indicated that the BPN–psychache association varied across levels of stress mindset (BPN × stress mindset: *b* = 0.106, *p* < 0.001). Figure 2 shows that the slope linking BPN to psychache remained negative at all levels of stress mindset but was steepest when stress mindset was low. Consistent with this pattern, the conditional direct effect weakened from −0.532 at low stress mindset to −0.321 at high stress mindset. Thus, greater BPN was associated with lower psychache across the sample, but this association was less pronounced among students who viewed stress as more enhancing.

**Figure 3:**
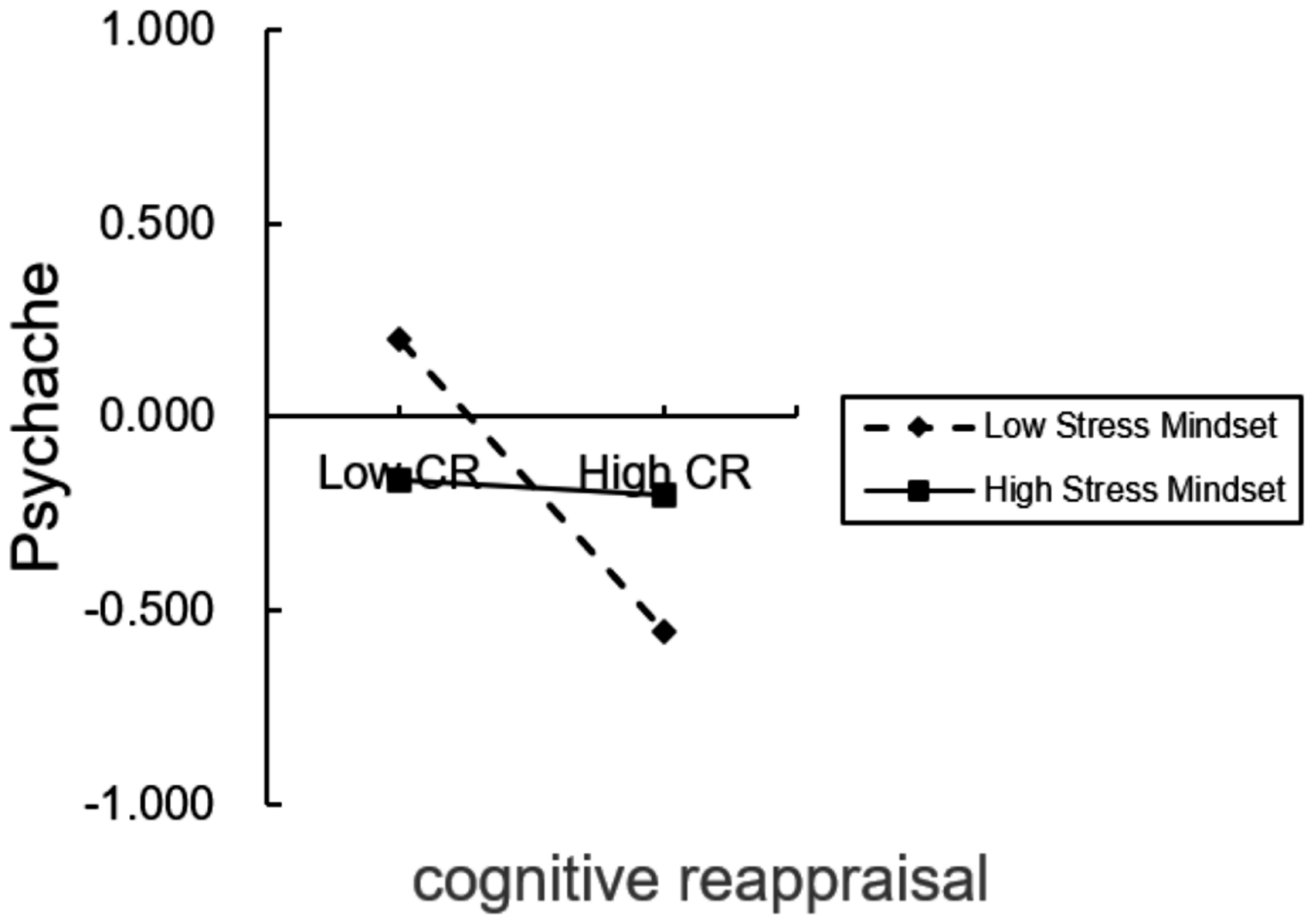
Conditional association between cognitive reappraisal (CR) and psychache at low (−1 SD) and high (+1 SD) levels of stress mindset.

A significant interaction between cognitive reappraisal and stress mindset indicated that the cognitive reappraisal–psychache association varied across levels of stress mindset (cognitive reappraisal × stress mindset: *b* = 0.054, *p* = 0.005). As illustrated in Figure 3, the simple slope for cognitive reappraisal was more strongly negative when stress mindset was low and became flatter as stress mindset increased. Correspondingly, the conditional indirect association through reappraisal was significant at low and average levels of stress mindset but smaller, and its 95% confidence interval included zero at high levels of stress mindset. These findings indicate a pattern of cross-sectional moderation rather than a protective mechanism.

Conditional total effects were significant across all levels of stress mindset, but their magnitude decreased from low to high stress mindset (TOT_LOW = −0.583, 95% CI [−0.642, −0.527]; TOT_MEAN = −0.456, 95% CI [−0.501, −0.412]; TOT_HIGH = −0.329, 95% CI [−0.385, −0.272]). The index of moderated mediation was statistically different from zero (IMM = 0.021, 95% CI [0.007, 0.036]), indicating that the estimated indirect association via reappraisal varied across levels of stress mindset, although the magnitude of this variation was modest.

**Figure 4:**
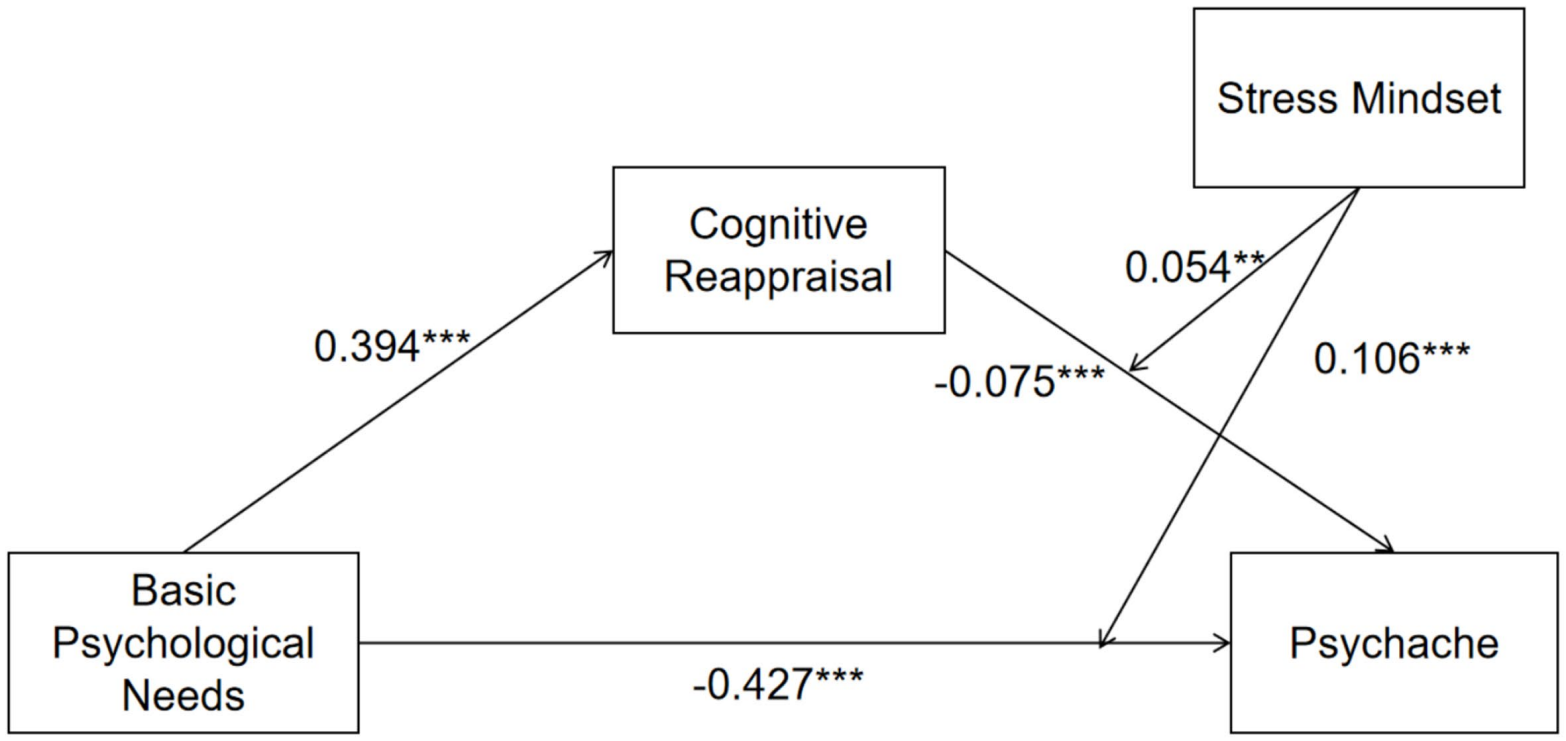
Conditional indirect association of BPN with psychache via cognitive reappraisal across levels of stress mindset.

Given the significant interaction between cognitive reappraisal and stress mindset, the association between cognitive reappraisal and psychache varied across levels of stress mindset. Specifically, the negative association between reappraisal and psychache was weaker at higher levels of stress mindset; therefore, Hypothesis 4 was supported (see Figure 4).

## 4 Discussion

Grounded in Self-Determination Theory and the process model of emotion regulation, this study examined how BPN, cognitive reappraisal, stress mindset, and psychache were related in a cross-sectional sample of medical students. Three findings stand out. First, higher BPN was associated with lower psychache. Second, cognitive reappraisal statistically accounted for a modest portion of this association. Third, the strength of both the direct BPN–psychache association and the reappraisal–psychache association varied across levels of stress mindset, such that the negative associations were weaker at higher levels of stress-is-enhancing mindset. These findings suggest that motivational resources, regulatory strategies, and stress-related beliefs may be considered together when studying psychache.

### 4.1 Basic psychological needs and psychache

The negative association between BPN and psychache is consistent with SDT, which posits that autonomy, competence, and relatedness are central to psychological adjustment (Deci and Ryan, 2004). In the present sample, students who reported greater fulfillment of these needs also reported less psychological pain. This pattern aligns with prior evidence linking need satisfaction to better mental health and lower distress (Neufeld and Malin, 2019; Bai et al., 2021). For medical students, whose training often involves persistent evaluation and role strain, unmet needs may coincide with feelings of helplessness, isolation, or inefficacy that are phenomenologically close to psychache.

At the same time, the effect should be interpreted with appropriate caution. The direct association remained robust but was not so large as to imply that BPN alone explains psychache, and the model’s *R*^2^ for psychache indicates that substantial variance remains unaccounted for. Other factors, such as depressive symptoms, academic overload, personality traits, or interpersonal stress, may also contribute importantly to psychache and may partly overlap with perceived need satisfaction.

### 4.2 Indirect association via cognitive reappraisal

Cognitive reappraisal reflected one statistical indirect pattern linking BPN and psychache. Students with higher BPN reported greater use of reappraisal, and greater reappraisal was associated with lower psychache, particularly when stress mindset was low or average. This pattern is compatible with the idea that need fulfillment and adaptive cognitive regulation may co-occur in ways that relate to lower emotional pain.

However, the indirect effect was modest and should not be overstated. In cross-sectional data, statistical mediation only indicates that the covariance among variables is consistent with an indirect pattern; it does not establish temporal order. It is equally plausible that students experiencing lower psychache perceive greater access to their psychological needs and report more frequent use of reappraisal, or that reciprocal relations operate over time. Accordingly, the present findings should be treated as hypothesis-generating rather than mechanistic evidence.

Specifically, higher BPN was associated with greater reported use of cognitive reappraisal (*a* = 0.394), and—conditional on stress mindset—greater reappraisal was associated with lower psychache. This pattern aligns with Gross’s process model (Gross, 2015) and SDT-informed accounts suggesting that need fulfillment may co-occur with greater use of adaptive cognitive strategies. Importantly, because all variables were measured at a single time point, these findings should be interpreted as cross-sectional associations (including statistical mediation) rather than evidence of temporal ordering or causal mechanisms.

### 4.3 Variation in associations across levels of stress mindset

Stress mindset emerged as a boundary condition of the observed associations. The negative association between BPN and psychache was weaker among students with a stronger stress-is-enhancing mindset, and the same attenuation pattern appeared for the reappraisal–psychache association. In descriptive terms, psychache was less strongly coupled to either BPN or reappraisal at higher levels of stress-is-enhancing mindset.

The attenuation of the indirect association at high stress mindset should be interpreted cautiously. It may reflect a weaker estimated role for reappraisal under those conditions, but alternative explanations are also plausible, including omitted variables, shared method variance, restricted range, model uncertainty, or reciprocal associations among the constructs. These alternatives should be tested in longitudinal and multi-method designs.

Importantly, the interaction effects were statistically reliable but modest in size. Thus, they are theoretically informative but should not yet be interpreted as strong evidence for tailoring interventions solely on the basis of stress mindset. Replication and stronger designs are needed before firm practical conclusions can be drawn.

### 4.4 Limitations

Several limitations should be acknowledged. First, the cross-sectional design does not permit conclusions about temporal precedence or causality. Although the proposed indirect pattern is theory-consistent, reverse or reciprocal associations remain plausible. Second, all variables were assessed via self-report at a single time point, which raises the possibility of shared method variance. Although procedural remedies and statistical diagnostics were applied, common method bias cannot be definitively ruled out. Third, participants were recruited from five medical schools in one Chinese province using a convenience-within-cluster strategy. The sample was therefore not only geographically restricted but also educationally homogeneous, because all participants were medical students. These features limit generalizability to broader student populations, other disciplinary or professional training settings, and more diverse cultural and educational contexts. Fourth, the model explained a modest proportion of variance, particularly for cognitive reappraisal (*R*^2^ = 0.159), and some statistically significant effects were modest in magnitude, which may constrain practical significance at the individual level. Fifth, although Self-Determination Theory distinguishes among autonomy, competence, and relatedness, the present analyses used a composite BPN score, which may have obscured dimension-specific associations. Finally, the model did not include other potentially important determinants of psychache, such as depressive symptoms, social support, academic stressors, and personality characteristics. Future research should incorporate these factors and use stronger designs to test temporal dynamics and boundary conditions more rigorously.

### 4.5 Implications

Despite these limitations, the present study may still offer a useful, theory-informed perspective on psychache in medical students. Specifically, the findings suggest that psychache may be better understood when basic psychological needs, cognitive reappraisal, and stress-related beliefs are considered jointly rather than in isolation.

These implications should be interpreted cautiously. Because the data are cross-sectional and self-reported, the results do not justify causal conclusions or intervention recommendations. Instead, they provide empirical estimates and theoretically relevant hypotheses for future longitudinal, experimental, and multi-method research.

### 4.6 Conclusion

In this cross-sectional sample of medical students, higher basic psychological need satisfaction was associated with lower psychache, and cognitive reappraisal statistically accounted for a modest portion of that association. The strength of both the direct and indirect associations varied across levels of stress mindset, with weaker negative links to psychache at higher levels of stress-is-enhancing mindset. These results should be interpreted as context-specific, theory-informed cross-sectional associations rather than evidence of temporal or causal mechanisms. Future longitudinal and experimental studies are needed to determine temporal ordering, rule out alternative explanations, and assess the practical significance of these associations.

## Data Availability

The raw data supporting the conclusions of this article will be made available by the authors, without undue reservation.
